# The Influence of Single Nucleotide Polymorphism Microarray-Based Molecular Karyotype on Preimplantation Embryonic Development Potential

**DOI:** 10.1371/journal.pone.0138234

**Published:** 2015-09-18

**Authors:** Gang Li, Nannan He, Haixia Jin, Yan Liu, Yihong Guo, Yingchun Su, Yingpu Sun

**Affiliations:** Reproductive Medical Center, First Affiliated Hospital of Zhengzhou University, Zhengzhou, China; State Key Laboratory of Reproductive Biology, Institute of Zoology, Chinese Academy of Sciences, CHINA

## Abstract

In order to investigate the influence of the molecular karyotype based on single nucleotide polymorphism (SNP) microarray on embryonic development potential in preimplantation genetic diagnosis (PGD), we retrospectively analyzed the clinical data generated by PGD using embryos retrieved from parents with chromosome rearrangements in our center. In total, 929 embryos from 119 couples had exact diagnosis and development status. The blastocyst formation rate of balanced molecular karyotype embryos was 56.6% (276/488), which was significantly higher than that of genetic imbalanced embryos 24.5% (108/441) (*P*<0.001). No significant difference was detected in blastocyst formation rates in the groups of maternal age<30, 30–35 and >35 respectively. Blastocyst formation rates of male and female embryos were 44.5% (183/411) and 38.8% (201/518) respectively, with no significant difference between them (*P*>0.05). The rates of balanced molecular karyotype embryos vary from groups of embryos with different cell numbers at 68 hours after insemination. The blastocyst formation rate of embryos with 6–8 cells (48.1%) was significantly higher than that of embryos with <6 cells (23.9%) and with >8 cells (42.9%) (P<0.05). As for the unbalanced embryos, there was no significant difference of the distribution of abnormal molecular karyotypes in the subgroup of the arrest, morula and blastocyst. Thus, we conclude that embryos with balanced molecular karyotype have significant higher development potential than those with imbalanced molecular karyotype whilst maternal age, embryo gender and types of abnormal molecular karyotype have no significant influence on blastocyst formation. Compared with embryos with <6 and >8 cells, embryos with 6–8 blastomeres have higher rate of balanced molecular karyotype and blastocyst formation.

## Introduction

Preimplantation genetic diagnosis (PGD) or screening (PGS) of biopsied embryos unveils embryonic chromosomal complement, which demonstrates that more than 50% of embryos contain chromosomally abnormal blastomeres. A host of studies have supported that embryos with abnormal karyotype have a lower cleavage rate than those with normal karyotype[1]; hence, it is hypothesized that extended culture to blastocyst stage may help to select embryos with normal karyotype and high implantation potential. Nevertheless, many studies have suggested that a large part of embryos with abnormal chromosome are still able to form blastocysts. As a result, extended culture cannot eliminate all chromosomally abnormal embryos [2]. Fluorescence *in situ* hybridization (FISH) is used to be performed in PGD but it has a couple of limitations. To begin with, it could analyze only 10 to 12 chromosomes and is not able to detect tiny chromosomal abnormality. Besides, failed single cell fixation may result in loss of signal and misdiagnosis. In recent years, new methods are used for PGD, such as single nucleotide polymorphism microarray (SNP microarray) technology which has a host of advantages compared to former methods. It is able to detect 23 pairs of chromosomes. Furthermore, its high resolution, ability of detecting small indels or duplication and less misdiagnosis make its diagnosis more reliable [3,4], and it is likely to improve the clinical outcome of PGD for translocation carriers [5,6]. However, to our knowledge, the impact of the molecular karyotype based on SNP microarray on embryo development has not been analyzed.

The aim of this study is to explore the relationship between molecular karyotype and embryonic development potential, which could be helpful to select embryos with higher development potential to transfer and provide foundation for enhancing the pregnancy rate.

## Materials and Methods

### Clinical case details and patient counseling

Between May 2011 and Sep 2012, a total of 119 couples performed 128 fresh PGD cycles in our reproductive medical center. All patients with structural chromosomal rearrangements were assessed by geneticists and reproductive endocrinologists. Genetic counseling involved reviewing the couple’s three-generation history and explaining in detail the PGD process, including the accuracy and limitations of the microarray PGD/PGS. Possible genetic outcomes, success rates, and risks of misdiagnosis were also discussed. The couples were then referred to our center to undergo standard IVF with 100% intracytoplasmic sperm injection (ICSI). All women enrolled in this study were documented to have normal ovarian reserve, as defined by a follicle stimulating hormone (FSH) level of less than 10 mIU/ml. All women enrolled in the study were documented to have no gynecologic abnormalities including anatomical defects/abnormalities. A written informed consent was obtained from all patients, in which the possible risk of misdiagnosis was specified and confirmatory prenatal diagnosis for any ensuring pregnancy was recommended. This work has been approved by the Institutional Review Board (IRB) of First Affiliated Hospital of Zhengzhou University.

### Notes on embryo biopsy and cell lysis

Cleavage-stage embryos were obtained using a standard ICSI procedure. Follicular aspiration was performed under transvaginal ultrasound guidance. Afterwards, mature oocytes were injected with a single spermatozoon approximately 4 hours after follicular aspiration. All Cleavage stage embryos were biopsied and underwent clinical microarray analysis. To accomplish this, embryo biopsy was performed using an OCTAX LasershotTM and one cell was removed from each embryo for genetic testing. Each single blastomere was placed into an eppendorf tube of 5 μl 0.2N potassium hydroxide (KOH) DNA stabilizing buffer.

### SNP microarray and data interpretation

All single cells were first subjected to a modified multiple displacement amplification protocol followed by a second round of whole genome amplification [7]. To accomplish this, the cells were first lysed using an alkaline denaturation buffer, taking care not to shear the DNA from lysed cells which could result in breaks in the genomic DNA. A modified multiple displacement amplification protocol was then employed utilizing *phi*29 DNA polymerase to complete the first round of DNA amplification. Four microliters (200ng) of the remaining multiple displacement amplified DNA then underwent another round of DNA amplification, using a modified whole genome amplification protocol. Approximately 200,000 ng of amplified DNA was loaded onto Illumina high-density HumanCytoSNP-12 DNA beadchips (Illumina, San Diego, CA) containing 301, 232 genetic markers and routine microarray analysis and scanning was performed by an Illumina HiScanSQ BeadArray reader. Bioinformatics was accomplished using Illumina GenomeStudio software. Clinical data was compared to an established embryonic cell normalized data set. Data from the Illumina system was resulted and interpreted to establish whether each embryo was normal or had a genomic imbalance associated with the parental translocation chromosomes or general aneuploidy. All final molecular karyotypic analysis was performed with the reader, a medical geneticist, blinded to patient names or controls. Clinical reports were generated and appropriate embryos were transferred.

### Statistical analysis

For analysis, SPSS12.0 software (version 9, SPSS Inc., Chicago, USA) was used. The results are expressed as the mean ± SD. The p values less than 0.05 were considered significant. Chi-square test was used to test the frequencies and Student's t-test was used for comparing the means.

## Results

**Fig 1:**
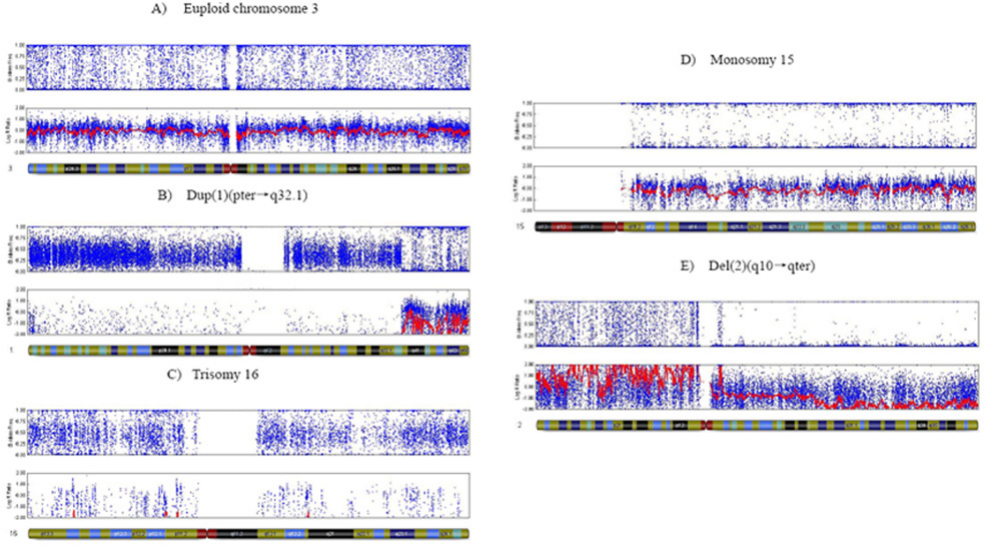
Molecular karyotyping of preimplantation embryo using 23-chromosome SNP Microarray. (A)demonstrates the normal diploid diagnostic reading obtained from a blastomere for chromosome 3. Normal AA, AB and BB alleles and a 0 reading for the smooth log *R* ratio is observed. (B)illustrates the duplication of pter→q32.1reading of chromosome 1 from a cleavage stage embryo. AA, AB and BB alleles are observed from q32.1 to qter of chromosome 1; however, AAA, AAB ABB and BBB are observed from pter to q32.1 of chromosome 1. A significant shift in the smooth log *R* ratio is observed from pter to q32.1 of chromosome 1. (C)shows a trisomy reading of chromosome 16, from a cleavage stage embryo. AAA, AAB ABB and BBB are observed without AB alleles represented. A significant shift in the smooth log *R* ratio is observed, consistent with the trisomy karyotype. (D)presents a monosomy reading of chromosome 15, from a cleavage stage embryo. AA and BB alleles are observed without AB alleles represented. A significant shift in the smooth log *R* ratio is observed, consistent with the monosomy karyotype. (E)displays the deletion of q10 to qter reading of chromosome 2 from a cleavage stage embryo. AA, AB and BB alleles are observed in pter to q10 of chromosome 2. However, AA and BB alleles are observed without AB in q10 to qter of chromosome 2 represented. A significant shift in the smooth log *R* ratio is observed in q10 to qter of chromosome 2.

119 couples underwent 128 fresh PGD cycles and mean maternal age was 30.35±4.50. A total of 1075 day-3 embryos were biopsied while 929 of which had exact diagnosis and were extendedly cultured to the blastocyst stage, 488 (52%) of which were molecular karyotype balanced embryos (Fig 1A). 384 blastocysts were formed and the total formation rate was 41.3%.

### The influence of molecular karyotype on embryonic development

**Fig 2:**
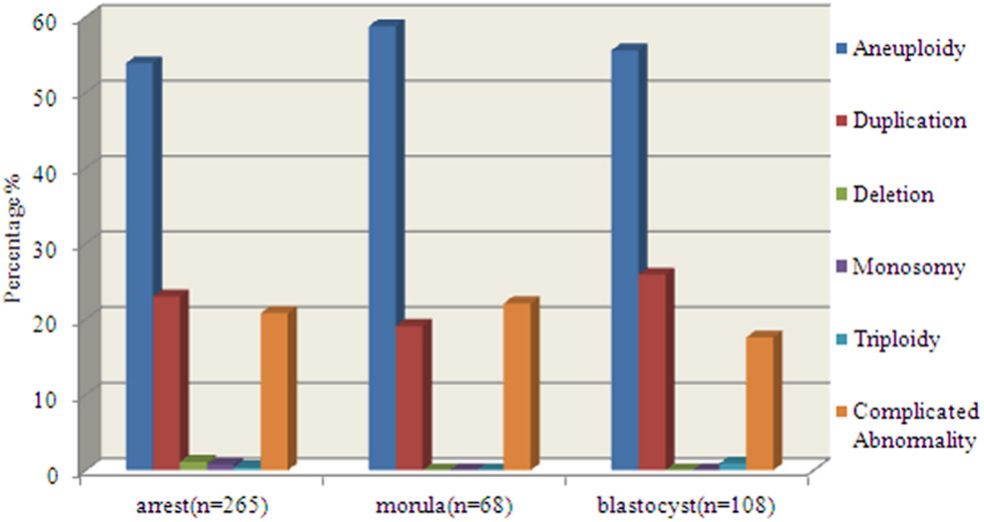
The distribution of abnormal molecular karyotypes in the subgroup of the arrest, morula and blastocyst. Blue represents the aneuploidy, red indicates the duplication and yellow indicates the complicated abnormalities and so on.

There was no significant difference (*P* = 0.224) between the mean maternal ages in two groups of molecular karyotype balanced and imbalanced embryos, which were 30.18±4.57 and 30.54±4.43 respectively. However, the blastocyst formation rate of molecular karyotype balanced embryos was 56.6%(276/488), which was significantly higher than that of imbalanced embryos 24.5%(108/441)*P*<0.05) (Table 1). In the unbalanced embryos, the most popular molecular karyotype was aneuploidy (55%, 243/441) followed by duplication (23%, 102/441), complex abnormalities(20%, 89/441), deletion(0.7%, 3/441), monosomy(0.5%, 2/441) and triploidy (0.5%, 2/441)., there was no significant difference among blastocyst formation rates of embryos with different types of imbalanced molecular karyotypes like aneuploidy(Fig 1C and 1D), duplication(Fig 1B), deletion(Fig 1E) and complex abnormalities. Moreover, according the embryonic development stage, we divided the samples into three groups: the arrest, morula and blastocyst, then the rate of different types of imbalanced molecular karyotypes was analyzed. And there was no significant difference of the distribution of abnormal molecular karyotypes in the subgroup of the arrest, morula and blastocyst (Fig 2).

**Table 1:** The influence of molecular karyotype on embryonic development.

Molecular karyotype	Maternal age(y) (Mean±SD)	Arrest %(n)	Morula %(n)	Blastocyst %(n)*	Total (n)
Balanced	30.18±4.57	40.9% (200/488)	2.5% (12/488)	56.6%(276/488)	488
Imbalanced	30.54±4.43	60.1% (265/441)	15.4% (68/441)	24.5%(108/441)	441
Total	30.35±4.50	50.1% (465/929)	8.6% (80/929)	41.3%(384/929)	929

* Chi-square test, *χ* ^2^ = 119.7, *P*<0.05

### The influence of maternal age and gender on blastocyst formation

No significant difference was detected among blastocyst formation rates of different female ages(*P* = 0.254). The blastocyst formation rates were 41.4%(196/473), 42.5%(119/280)and 39.2%(69/176) in the group of maternal age <30, 30–35 and >35 years old respectively. Similarly, no significant difference was detected among blastocyst formation rates of different female ages both in groups of embryos with balanced and imbalanced molecular karyotype(*P* = 0.311 and 0.072 respectively, S1 Table).

The blastocyst formation rates of XY and XX embryos were 44.5%(183/411) and 38.8%(201/518)respectively, with no significant difference between them(*P* = 0.061). In similar, no significant difference was detected between blastocyst formation rates of male and female embryos both in groups of embryos with balanced and imbalanced molecular karyotype(*P* = 0.172 and 0.713 respectively, S2 Table).

### The relationship among blastomere number, embryonic development and molecular karyotype

**Fig 3:**
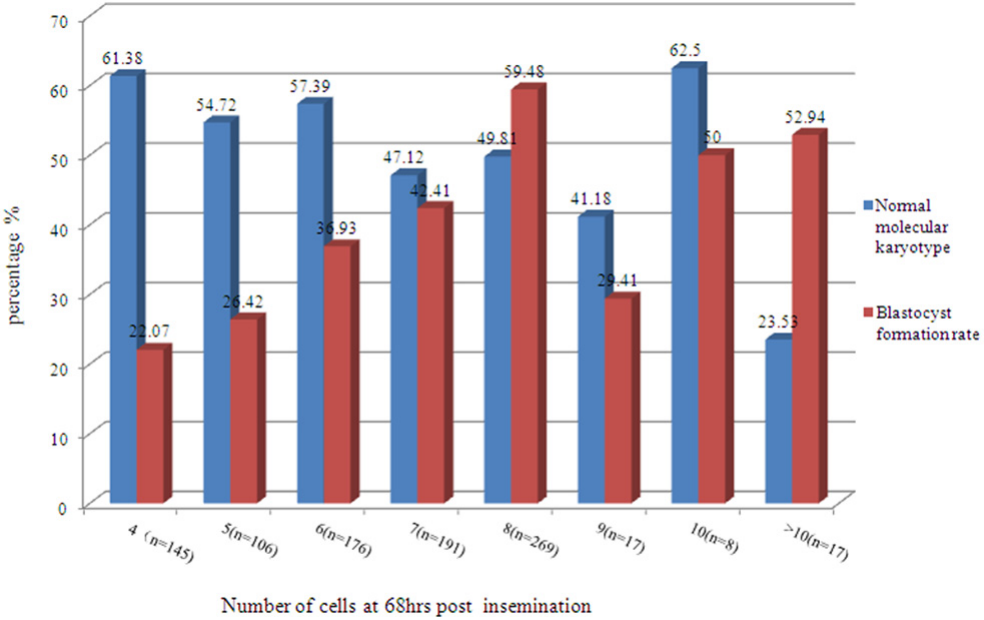
Normal molecular karyotype rate and blastocyst formation rate of embryos with different blastomere numbers. Blue represents normal molecular karyotype rate whilst red indicates the blastocyst formation rate.

The blastocyst formation rate of embryos with 6–8 cells (48.1%) was significantly higher than that of embryos with <6 cells (23.9%) and with >8 cells(42.9%) (*P*<0.001, respectively) (Table 2). And the rates of balanced molecular karyotype of subgroup with <6 and 6–8 cells were 58.6%(147/251)and 51.1%(325/636),which were significantly higher than that of embryos with >8 cells 38.1%(16/42) (*P* = 0.045 and 0.013, respectively). The rates of balanced molecular karyotype and blastocyst formation vary among the subgroup which divided by different cell number at 68hrs post insemination (Figs 1 and 3).

**Table 2:** The relationship among blastomere number 68hr after fertilization, embryonic development and molecular karyotype.

Blastomere number	<6	6–8	>8	*P* value
Blastocyst formation rate of embryos with balanced molecular karyotype %(n)	32.0%(47/147)	67.4%(219/325)	62.5%(10/16)	0.001
Blastocyst formation rate of embryos with imbalanced molecular karyotype %(n)	12.5%(13/104)	28%(87/311)	30.8%(8/26)	0.005
Total Blastocyst formation rate %(n)	23.9%(60/251)	48.1%(306/636)	42.9%(18/42)	0.001

## Discussion

### The influence of molecular karyotype on embryonic development

Fluorescence *in situ* hybridization (FISH) and comparative genomic hybridization (CGH) were introduced previously to study the influence of genetic aberrance on embryonic development. However, to our knowledge, the impact of the molecular karyotype based on SNP microarray on embryo development has not been analysed. Furthermore, compared with the FISH and CGH, the SNP microarray technology used in this research has numerous merits [8,9]. In the first place, it is able to detect whole 23 pairs chromosomes. In the second place, the diagnosis was more reliable with the higher resolution and the ability of detecting small indels or duplication.

Our data show that the blastocyst formation rate of balanced molecular karyotype embryos was 56.6%, which was significantly higher than that of imbalanced embryos 24.5%(*P*<0.001). It is consistent with several previous studies performed by FISH and CGH. Sandalinas et al analyzed the karyotype of 254 day-3 embryos using FISH, suggesting that the blastocyst formation rate of chromosomally normal embryos (66%) was significantly higher than that of chromosomally abnormal embryos (15%) [10].Magli et al analyzed the karyotype of 143 day-3 embryos using FISH, demonstrating that aneuploid embryos were much more likely to arrest and had a lower blastocyst formation rate than euploid embryos [11]. What is more, although Ribeiro et al found no significant difference between the blastocyst formation rate of aneuploid embryos and euploid embryos after karyotype analysis of 88 day-3 embryos, it was generally accepted that chromosomal abnormality could reduce embryonic development potential, resulting in slow cleavage rate and low blastocyst formation rate [2].

In addition, our data show that embryos with balanced molecular karyotype had significant higher blastocyst formation rate but a quarter of embryos with imbalanced molecular karyotype were still able to form blastocysts. It means extended culture to blastocyst stage could not eliminate all molecular karyotype abnormal embryos, although a certain part of which could be prevented by embryonic densification. Moreover, there was no significant difference among blastocyst formation rates of embryos with different types of imbalanced molecular karyotypes like aneuploidy, duplication, deletion and complex abnormalities.

### The influence of maternal age on blastocyst formation

It is generally accepted that female fertility decreases with increasing age. The decline in pregnancy rate of older female patients in assisted reproduction can be due to several factors, such as low oocytes count, decreased quality of eggs or embryos and poor uterine receptivity. Most of the findings about the influence of maternal age on embryo quality demonstrated that embryonic development potential decreased with increasing maternal age. Janny et al found that embryos from elderly women had a lower blastocyst formation rate and expansion degree [12]. Similarly in 1999, Kostas et al extendedly cultured 3115 embryos to day 5/6 and their study showed that blastocyst formation rate of embryos with maternal age ≥ 40 years old was 22.2%, which was significantly lower than that of embryos with maternal age <40 years old [13]. The result of research performed by Bruce et al, who analyzed the relationship between maternal age and blastocyst formation, showed that the blastocyst formation rate gradually decreased with increasing maternal age [14]. On the contrary, Michael et al found in their research that the blastocyst formation rate depended on retrieved follicles and patients’ ovarian ages, rather than their biological ages [15].

However, our results show that there was no significant difference in blastocyst formation rate between embryos with different maternal age. What should be mentioned here is that our study is about embryonic development after biopsy. Considering the fact that cell biopsy may affect the embryonic development and in our study there were only 131 embryos from women > 35 years old and 17 embryos from women≥ 40 years old, which was a small sample size, we may not conclude that maternal age does not affect the embryonic blastocyst formation rate.

### The influence of gender on embryonic development potential

In terms of the influence of gender on embryonic development, up to now animal experimental data support that male embryos had a higher blastocyst formation rate than female embryos whist there are a few studies on human embryos and the results are inconsistent. Some studies have shown that the human male cleavage stage embryos and blastocysts have a larger number of cells than female embryos. Besides, blastocyst transfer may increase male infant birth rate [16]. Samer et al analyzed the trophectodermal chromosomal status of 500 blastocysts using CGH in order to compare the development and day-5 morphology between male and female embryos [17]. Eventually, their study showed faster development and a high blastocyst score in male embryos. Nevertheless, Eaton et al found no significant difference in blastocyst formation rate between male and female embryos after they used FISH to analyze the chromosomal complement of 143 day-3 embryos [18].Our results showed that in the group of embryos with balanced molecular karyotype, male embryos had a higher blastocyst formation rate (59.3%) than female embryos (54.0%) although there was no significant difference (*P* = 0.172), suggesting that embryonic gender had no significant influence on blastocyst formation rate.

### The relationship among blastomere number, embryonic development and molecular karyotype

It is ideal that embryos have 8 blastomeres three days after fertilization. Many studies have explored the influence of cell number on embryonic genetic status and development. And the conclusions were inconsistent.

Some data has shown that 8-cell embryos on D3 have the lowest rate of chromosomal abnormalities. M. Cristina Magli et al have demonstrated that 4665 embryos were selected for chromosomal analysis by means of FISH after single cell biopsy 62 hours after insemination. It was illustrated that the incidence of chromosomal abnormalities was significantly higher in arrested or slow-cleaving embryos, and in embryos cleaving too fast, compared to embryos with eight cells at 62 hours after insemination, which had the lowest chromosomal abnormalities of 48% [19]. Similarly, Bielanska et al found that aneuploid embryos developed slower [20].

However, other studies suggested that there was no relation between cleavage speed and chromosomal status. Ziebe et al found that embryos with less than 4 cells did not have a higher aneuploidy rate than those with more than 4 cells 68±1 hours after fertilization [21]. It can be seen from our study that 58.6%(147/251)and 51.1%(325/636)of embryos with less than 6 and 6–8 cells were chromosomally normal respectively, which were significantly higher than that of embryos with more than 8 cells38.1%(16/42) (*P* = 0.045 and 0.013, respectively).

Besides, some studies have shown that embryos with more cells have a higher blastocyst formation rate. Bruce S. et al found that embryonic blastocyst formation rate increased with cell number from 4 to 8 [22]. Besides, embryos with more cells were more likely to form expanded blastocysts while embryos with less than 5 cells hardly formed blastocysts. In Luna M’s study, there was no significant difference in blastocyst formation rate between embryos with 7–9 cells and embryos with no less than 10 cells, both of which were significantly higher than that of embryos with no more than 6 cells [23].

Our data indicate that the blastocyst formation rate of embryos with 6–8 cells (48.1%) was significantly higher than that of embryos with less than 6 cells (32.0%) and with more than 8 cells (42.9%) (*P*<0.05, respectively). Moreover, blastocyst formation rate did not increase gradually with cell numbers of embryos. Furthermore, balanced molecular karyotype rate and blastocyst formation rate were also analyzed among embryos with different cell numbers, from 4 to more than 10 cells. Embryos with 4, 5 and 6 cells had higher balanced molecular karyotype rate than 8-cell embryos. However, the blastocyst formation rate of 8-cell embryos was significantly higher than that of embryos with 4, 5, 6 and 7 cells (*P*<0.001). Consequently, it can be concluded that 8-cell embryos have a higher development potential.

There are also some limitations in the present study. Like other related clinical trials, one important limitation in our study was the retrospective design. In addition, sample size in some subgroup was relatively small; thus, some bias may exist in our analysis. Another limitation was that we only analyzed the molecular karyotype which could impact the embryo development. In fact, there were many other factors which could influence the development of embryo, such as culture environment, etc. More importantly, we used morphology criteria rather than implantation rate and clinical pregnancy rate to evaluate the development of embryos, which may not represent the ‘real potential’ of embryo development.

To sum up, embryos with balanced molecular karyotype based on SNP microarray analysis had higher development potential than those with imbalanced molecular karyotype. In contrast, maternal age, embryo gender and types of imbalanced molecular karyotype have no significant influence on development and blastocyst formation of embryos. In addition, embryos with<6 and 6–8 blastomeres have higher balanced molecular karyotype rate than those of embryos with >8 cells, and blastocyst formation rate of embryos with 6–8 cells was significantly higher than that of embryos with less than 6 cells and more than 8 cells. To our knowledge, this is the first and the largest comprehensive trial conducted evaluating the influence of 24-chromosome molecular karyotype on embryonic development.

## Supporting Information

S1 File Data for our manuscript. (DOC)

S1 Table The influence of maternal age on embryonic development. (DOC)

S2 Table The influence of embryonic gender on embryonic development. (DOC)
