# Supplementary material for: The Influence of Single Nucleotide Polymorphism Microarray-Based Molecular Karyotype on Preimplantation Embryonic Development Potential
Source: PLoS One. 2015 Sep 18;10(9):e0138234. doi: 10.1371/journal.pone.0138234 (PMC4575173; doi:10.1371/journal.pone.0138234)
Supplement: S1 File — (DOC) [file pone.0138234.s001.doc]

**Original data**

| *D3* | *Arrest* | *Morula* | *Blastocyst* | **Total** |
| --- | --- | --- | --- | --- |
| Balanced | 200 | 12 | 276 | 488 |
| Imbalanced | 265 | 68 | 108 | 441 |
| Total | 465 | 80 | 384 | 929 |

From May, 2011 to September, 2012.

**Balanced embryo**

| Age (Y) | Arrest | Morula | Blastocyst | Total |
| --- | --- | --- | --- | --- |
| ≤30 | 201 | 46 | 39 | 416 |
| 30-35 | 140 | 21 | 119 | 280 |
| ≥35 | 97 | 10 | 69 | 176 |

**Imbalanced embryo**

| Age (Y) | Arrest | Morula | Blastocyst | Total |
| --- | --- | --- | --- | --- |
| ≤30 | 120 | 43 | 59 | 222 |
| 30-35 | 84 | 18 | 28 | 130 |
| ≥35 | 61 | 7 | 21 | 89 |

**Total embryos**

| *Age (Y)* | *Arrest* | *Morula* | Blastocyst | *Total* |
| --- | --- | --- | --- | --- |
| ≤30 | 228 | 49 | 196 | 473 |
| 30-35 | 140 | 21 | 119 | 280 |
| ≥35 | 97 | 10 | 69 | 176 |
| Total | 465 | 80 | 384 | 929 |

Total embryos

| *Gender* | *Arrest* | *Morula* | Blastocyst | *Total* |
| --- | --- | --- | --- | --- |
| Male | 201 | 27 | 183 | 411 |
| Female | 264 | 53 | 201 | 518 |
| Total | 465 | 80 | 384 | 929 |

Balanced embryo

| *Gender* | *Arrest* | *Morula* | Blastocyst | *Total* |
| --- | --- | --- | --- | --- |
| Male | 93 | 3 | 140 | 236 |
| Female | 107 | 9 | 136 | 252 |
| Total | 200 | 12 | 276 | 488 |

**Imb**alanced embryo

| *Gender* | *Arrest* | *Morula* | Blastocyst | *Total* |
| --- | --- | --- | --- | --- |
| Male | 108 | 24 | 43 | 175 |
| Female | 157 | 44 | 65 | 266 |
| Total | 265 | 68 | 108 | 441 |

**Normal molecular karyotype rate and blastocyst formation rate of embryos with different blastomere numbers**

| D3 blastomere number | Balanced embryo proportion | D5blastocyst formation rate |
| --- | --- | --- |
| 4-cell | 89/145 | 32/145 |
| 5-cell | 58/106 | 28/106 |
| 6-cell | 101/176 | 65/176 |
| 7-cell | 90/191 | 81/191 |
| 8-cell | 134/269 | 160/269 |
| 9-cell | 7/17 | 5/17 |
| 10-cell | 5/8 | 4/8 |
| >10-cell | 4/17 | 9/17 |
