# Supplementary material for: The Influence of Single Nucleotide Polymorphism Microarray-Based Molecular Karyotype on Preimplantation Embryonic Development Potential
Source: PLoS One. 2015 Sep 18;10(9):e0138234. doi: 10.1371/journal.pone.0138234 (PMC4575173; doi:10.1371/journal.pone.0138234)
Supplement: S1 Table — (DOC) [file pone.0138234.s002.doc]

**The influence of maternal age on embryonic development**

| Maternal age(y) | <30 | 30-35 | >35 | *P* value |
| --- | --- | --- | --- | --- |
| Blastocyst formation rate of embryos with balanced molecular karyotype %(n) | 54.3%(120/221) | 57.7%(116/201) | 60.6%(40/66) | 0.311 |
| Blastocyst formation rate of embryos with imbalanced molecular karyotype %(n) | 25.4%(48/189) | 24.6%(46/187) | 21.5%(14/65) | 0.072 |
| Total Blastocyst formation rate %(n) | 41%(168/410) | 41.8%(162/388) | 41.2%(54/131) | 0.291 |
