# Supplementary material for: The Influence of Single Nucleotide Polymorphism Microarray-Based Molecular Karyotype on Preimplantation Embryonic Development Potential
Source: PLoS One. 2015 Sep 18;10(9):e0138234. doi: 10.1371/journal.pone.0138234 (PMC4575173; doi:10.1371/journal.pone.0138234)
Supplement: S2 Table — (DOC) [file pone.0138234.s003.doc]

**The influence of embryonic gender on embryonic development**

| Embryonic gender | Male | Female | *P* value |
| --- | --- | --- | --- |
| Blastocyst formation rate of embryos with  balanced molecular karyotype %(n) | 59.3%(140/236) | 54.0%(136/252) | 0.172 |
| Blastocyst formation rate of embryos with  imbalanced molecular karyotype %(n) | 24.6%(43/175) | 24.4%(65/266) | 0.713 |
| Total Blastocyst formation rate %(n) | 44.5%(183/411) | 38.8%(201/518) | 0.061 |
